# Mode of action of maintenance immunotherapy therapy with the TLR-9 agonist MGN1703 in metastatic colorectal carcinoma: the Phase III IMPALA study

**DOI:** 10.1186/2051-1426-3-S2-P173

**Published:** 2015-11-04

**Authors:** Alexander Stein, Kerstin Kapp, Werner Scheithauer, Ramon Salazar, Michel Ducreux, Tom Waddell, Dirk Arnold, Christophe Tournigand, Alberto Sobrero, Eric Van Cutsem, Manuel Schmidt, David Cunningham

**Affiliations:** 1 University Cancer Center Hamburg, Hamburg, Germany; 2 Mologen AG, Berlin, Germany; 3 Medical University of Vienna, Vienna, Austria; 4 Institut Català D´Oncologia, Barcelona, Spain; 5 Gustave Roussy Cancer Campus Grand Paris, Villejuif, France; 6 Royal Marsden Hospital, London, UK; 7 Klinik für Tumorbiologie, Freiburg, Germany; 8 Hôpital Henri Mondor, Créteil, France; 9 IRCCS Ospedale San Martino IST, Genova, Italy; 10 University Hospitals Gasthuisberg, Leuven, Belgium

## Background

MGN1703, a potent immunomodulatory Toll-like receptor 9 (TLR-9) agonist, was compared to placebo in the previous double-blind, randomized Phase II IMPACT study. The drug was given as switch maintenance in 59 patients with metastatic colorectal cancer (mCRC) not progressing after 4.5 to 6 months of induction chemotherapy +/- bevacizumab, considered the most appropriate setting for an agent broadly activating the immune system. MGN1703 showed a superior effect over placebo, with a hazard ratio (HR) for the primary endpoint “PFS on maintenance” of 0.55 (p=0.041) by local assessment. Exploratory PFS analyses of pretreatment characteristics identified patients with objective RECIST response, normalized CEA and presence of activated NKT cells at the end of induction treatment to benefit the most with MGN1703 maintenance. Furthermore, the subgroup of patients with RECIST response after induction therapy reported a HR of 0.40 (median 24.5 vs. 15.1 months) for overall survival (OS). These results warranted confirmation in a larger Phase III study.

## Methods

The IMPALA study (NCT02077868) is an open-label Phase III trial with participation of AIO, TTD, and GERCOR cooperative groups, designed to confirm the encouraging evidence from IMPACT. mCRC patients having achieved an objective tumor response following first line induction therapy with or without biological agents are randomized to receive MGN1703 switch maintenance monotherapy in the experimental arm or local standard of care in the control arm. In case of relapse, patients will reintroduce the initial induction treatment whenever feasible, with those in the experimental arm continuing to receive MGN1703 therapy. The primary endpoint of the study will be OS. Secondary endpoints include PFS, response rates, safety, and QoL in selected centers. Type of induction treatment, CEA and activated NKT at baseline are stratification factors for the study and will be prospectively assessed. All randomized patients take part in a comprehensive immune monitoring plan evaluating cytokines and chemokines in serum and the activation status of various immune cell populations. Recruitment started in September 2014 and the study is expected to recruit 540 patients within 24 months in 8 European countries.

After more than 100 patients have been recruited, first exploratory immune monitoring data of patient's blood samples confirmed the MoA of MGN1703: A strong activation was observed in monocytes (CD169+), a lesser one in NK cells (CD69+), a moderate activation in NKT cells and T cells (both CD69+). This immune profile is in keeping with data obtained from the previous IMPACT study.

## Trial registration

ClinicalTrials.gov identifier NCT02077868.

